# Theoretical Design of Smart Bionic Skins with Self-Adaptive Temperature Regulation

**DOI:** 10.3390/ma17225580

**Published:** 2024-11-15

**Authors:** Yubo Wang, Yungui Ma, Rui Chen

**Affiliations:** 1 Centre for Optical and Electromagnetic Research, State Key Lab of Modern Optical Instrumentation, College of Optical Science and Engineering, Zhejiang University, Hangzhou 310027, China; wybwang@zju.edu.cn (Y.W.); yungui@zju.edu.cn (Y.M.); 2 International Research Center for Advanced Photonics, Zhejiang University, Haining 314400, China

**Keywords:** phase transition, self-adaptive temperature modulation, VO_2_, bionic skin, multiband spectral control

## Abstract

Thermal management presents a significant challenge in electric design, particularly in densely packed electronic systems. This study proposes a theoretical model for radiative bionic skin that emulates human skin, enabling the self-adaptive modulation of the thermal exhaustion rate to maintain homeostasis for objects covered by the skin in fluctuating thermal environments. The proposed artificial skin consists of phase change material (VO_2_) nanoparticles embedded in a low-loss matrix situated on a metallic substrate with a minimal thickness of several micrometers. The findings from our theoretical analyses indicate that substantial alterations in thermal radiation power around the phase transition temperature of 340 K enable a silicone substrate to sustain a relatively stable temperature, with variations confined to ±6 K, despite external heat fluxes ranging from 150 to 450 W/m^2^. Furthermore, to improve the spectral resemblance to natural skin, a plasmonic surface composed of self-assembled silver nanocubes is incorporated, allowing for modifications to the visible light properties of the bionic skin while maintaining its infrared characteristics. This theoretical investigation offers a cost-effective and conformal approach to the design of ultra-compact, fully passive, and versatile thermal management solutions for robotic systems and related technologies.

## 1. Introduction

Thermal radiation plays a crucial role in various facets of the infrared spectrum, particularly in the areas of thermal management [1,2,3], camouflage [4], sensing [5], and thermography [6,7]. In this context, smart materials, which exhibit variations in emissivity in response to external stimuli, enable the dynamic control of thermal radiation. The ability to self-regulate temperature is of significant importance in applications where maintaining a stable temperature is essential, such as in robotic systems that must operate effectively in varying environmental temperatures. Consequently, the aim of this study is to develop a smart structure that emulates the self-adaptive temperature regulation found in biological skin. Additionally, the optical properties of the engineered bionic skin can be adjusted to facilitate multiband spectral control.

Thermochromic substances are among the most extensively studied smart materials, and they can either retain or release infrared thermal radiation energy when subjected to thermal stimuli, often involving phase transitions. For example, GST alloys [8,9,10] have been shown to be rapid, durable, and reproducible non-volatile phase transition materials. The transition between the cGST (crystalline phase) and aGST (amorphous phase) occurs gradually, resulting in an intermediate state that contains both amorphous and crystalline phases. By manipulating these intermediate phases, it is possible to create a continuously tunable thermal emitter within a specific temperature range [11]. Perovskite manganese oxides constitute another category of typical thermochromic material, one capable of transforming from ferromagnetic metals into paramagnetic insulators as temperature increases [12]. Relevant compositions have been synthesized to achieve tunable infrared properties [13]. Yu et al. introduced a design characterized by a transition temperature that decreased to 9 °C, alongside an emissivity variation from 0.23 to 0.73 within a temperature range of −50 to 50 °C [14]. This design meets the majority of thermal control requirements for spacecraft and is anticipated to be further optimized for more significant applications in the future. Furthermore, VO_2_ undergoes a transition from metallic to semiconducting behavior at 68 °C, a phenomenon first identified in 1959 and referred to as the metal–insulator transition [15]. This transition involves a structural alteration in VO_2_ from a monoclinic phase at lower temperatures to a rutile phase at higher temperatures. Importantly, this reversible transition can be induced not only by temperature fluctuations but also by mechanical stress, electric current, voltage, and laser photons (whether continuous-wave or pulsed). The adaptability of VO_2_ has led to extensive research into its properties, as it holds potential for a wide range of applications, including in optical and electrical switches [16], modulators [17], sensors [18], and memory devices [19].

The dynamic emissivity characteristics of VO_2_-based devices are significantly influenced by their structural configurations. Specifically, VO_2_ that has been deposited on high-emissivity substrates, such as SiO_2,_ exhibits negative differential emissivity, which facilitates greater energy emissions at lower temperatures and diminished emissions at higher temperatures [20,21]. In contrast, when VO_2_ is deposited on high-reflectance substrates such as Al, Au, and Ag, it demonstrates a positive differential emissivity performance. Consequently, the choice of device structure delineates the applications of VO_2_ into two distinct scenarios. Negative differential thermal emissivity can assist in maintaining the consistency of infrared signals with the surrounding environment, thereby enabling infrared dynamic concealment. Conversely, the positive differential thermal emissivity properties of VO_2_ enhance energy emission at elevated temperatures while inhibiting energy dissipation at lower temperatures [22,23]. Thus, the positive differential thermal emissivity of VO_2_-based devices can sustain objects at temperatures close to the critical temperature, aligning with our design objective of effective smart thermal management. Wu et al. developed a square array of silicon microcones. These were coated with a conformal layer of VO_2_. This design is advantageous due to the strong antireflective properties of cone arrays, which exhibit relative insensitivity to the angle of incidence, rendering them suitable for applications in thermal absorption and emission. The proposed thermal homeostasis structure demonstrated a nearly constant temperature response of 12 K, representing a 20-fold reduction in temperature variation compared to a silicon film [24]. However, the complexity of this structure poses challenges in terms of fabrication and cost-effectiveness. Furthermore, Shrewsbury et al. created a VO_2_-based multilayer structured film, achieving temperature fluctuations of approximately 25 K [25]. This approach is compatible with multilayer growth methods, facilitating large-scale production. However, it is essential to consider the response to visible light in order to better emulate the characteristics of bionic skins. In this study, we present a cost-effective and efficient approach to the development of smart bionic skins capable of self-adaptive temperature regulation. This is achieved through the utilization of a hybrid film composed of VO_2_ nanoparticles embedded within a polyethylene (PE) matrix. Unlike existing designs that rely on intricate geometric structures and which are limited to infrared spectrum responses, our single-layer hybrid film not only self-regulates temperature but also alters its color within the visible spectrum, making it suitable for large-scale applications in bionic robotic systems.

## 2. Materials and Methods

**Figure 1:**
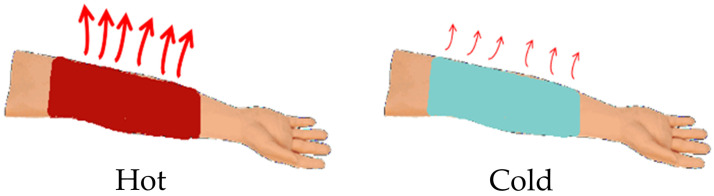
An illustration of self-adaptive temperature regulation. The red and blue arm regions covered by the smart skin coat represent the situations with high and low external heat loads, respectively. The red arrows denote the thermal radiation process, with the arrow thickness representing radiation strength. The radiation is automatically strengthened (weakened) with a high (low) heat load.

The designed functionalities are vividly elaborated in Figure 1. The object’s temperature can be automatically regulated to retain desired values by switching on or off the radiation of the bionic skin. Its bi-state infrared properties are numerically optimized by controlling the concentration and thickness through particle swarm optimization (PSO). With such a flexible coat, we show that an object made of a 500 μm silicone substrate can maintain constant designed homeostasis at temperatures around 340 ± 6 K under a heat pump whose power varies in the range of 150–450 W/m^2^. The temperature fluctuation seen is more than 5 times weaker than that observed the case of a pure VO_2_ film. Using this framework, we also show that a plasmonic surface made of self-assembled silver nanostructures can be introduced to freely tune the visible spectral appearance of the bionic skin to fulfill obtain the desired functions, with well-controlled multiband spectra ranging from infrared to visible light.

**Figure 2:**
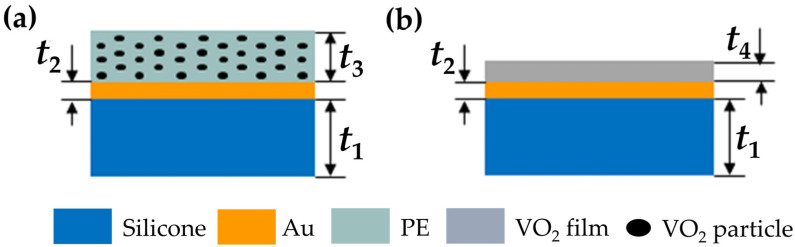
Schematics of the designed structures. (**a**) The designed structure composed of a hybrid film (mixture of VO_2_ nano-inclusions in the PE matrix) and a gold back plate. (**b**) The reference structure is composed of a pure VO_2_ film and a gold back plate. The substrate in both samples is made of silicone. The thicknesses for each layer are *t*_1_ = 500 μm, *t*_2_ = 0.2 μm, *t*_3_ = 1.13 μm, and *t*_4_ = 0.41 μm, respectively.

Figure 2 shows the design diagram. Here, we use a hybrid film composed of the PE matrix and VO_2_ nanoparticles as the main components of the smart bionic skins. The filling factor (*f*) and the film thickness (*t*_3_) are the key design parameters and are optimized through PSO. Further discussions are given below. A 200 nm (*t*_2_) thick gold background is utilized to reduce the backwards emissions (Figure 2a, hybrid film structure). For comparison, we also design a reference made of a pure VO_2_ film at an optimized thickness *t*_4_ (Figure 2b, VO_2_ film-based skin).

**Figure 3:**
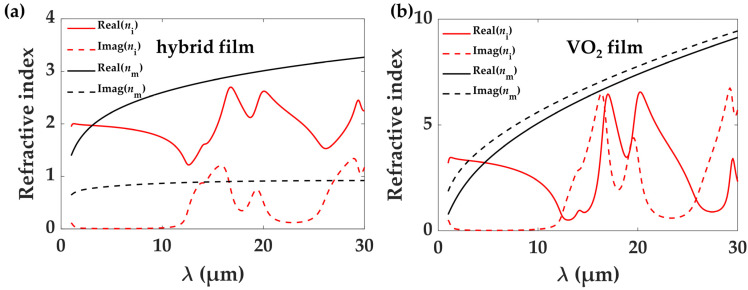
Effective index parameters for (**a**) hybrid film and (**b**) pure VO_2_ film. The solid lines are the real part of the refractive index and the dashed lines denote the imaginary part of the index. The red and black colors represent the insulating and metallic phase states of VO_2_, respectively.

Before discussing the properties of the proposed structure, we firstly calculate the equivalent index of the hybrid film. Considering the mixture structure and the filling factor, in this paper, we use the Bruggeman model [26,27] to calculate the equivalent index at the static limit approximation in this paper:

(1) $$f\frac{\varepsilon_{1}-\varepsilon_{\mathrm{eff}}}{\varepsilon_{\mathrm{eff}}+p\left(\varepsilon_{1}-\varepsilon_{\mathrm{eff}}\right)}+(1-f)\frac{\varepsilon_{2}-\varepsilon_{\mathrm{eff}}}{\varepsilon_{\mathrm{eff}}+p\left(\varepsilon_{2}-\varepsilon_{\mathrm{eff}}\right)}=0,$$

where *ε*_1_, *ε*_2,_ and *ε*_eff_ are the permittivity of medium 1, medium 2, and the composite material, respectively, *f* is the filling factor of medium 1, and *p* is the depolarization factor. In this paper, medium 1 and 2 denote PE and VO_2_, respectively, where *p* = 1/3 and *f* is a variable (*f* = 0.3 is selected in Figure 3a). For PE, we use a constant refractive index *n*_PE_ = 1.5 − i(1.6 × 10^−3^) in our calculations. As for VO_2_, when it is in the insulating state, it can be described using a classical harmonic oscillator model, and in the metallic state, a Drude model can be used to characterize its permittivity (Figure 3b) [28]. The calculated results are shown in Figure 3a. From these, we can see that the attenuation coefficient of the hybrid film is high in the metallic state and low in the insulating state, especially in the 2.5–11 μm range. At longer wavelengths, the polar phononic resonance of the dielectric VO_2_ increases the loss.

**Figure 4:**
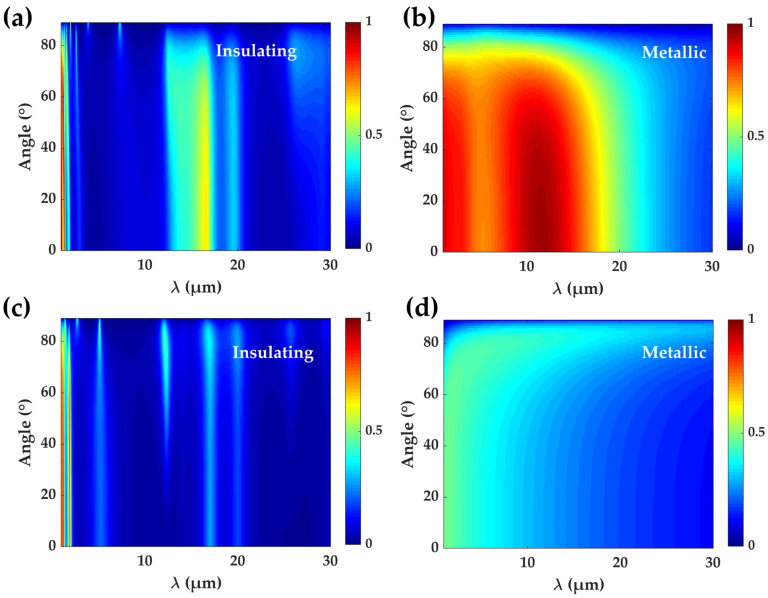
Infrared spectra emissivity of the samples at different wave emission angles. (**a**,**b**) The emissivity of the hybrid film structure with insulating and metallic VO_2_, respectively. (**c**,**d**) The emissivity of the reference VO_2_ film structure with insulating and metallic VO_2_, respectively. Colors represent the value of emissivity.

Next, we calculate the infrared spectra emissivity of the two structures mentioned above at different light emission angles using the transfer matrix theory, considering both s-polarization and p-polarization. The analytical results are presented in Figure 4. For the hybrid film, obviously, the emissivity values at two phases states of VO_2_ are quite different (Figure 4a,b). Although the emissivity with metallic VO_2_ decreases at larger emission angles, it still keeps a high value larger than 0.7 in the range of 2.5–13.8 μm up to an angle of 68.5°. When VO_2_ turns into the insulating phase, the emissivity is substantially suppressed, except for at some specific wavelength points (high emissivity is caused by the increased imaginary parts of the index, as shown in Figure 3a). For the reference sample, i.e., a pure VO_2_ film, the calculated results, shown in Figure 4c,d, indicate that the emissivity in the insulating state is still low but that, in the metallic state, the emissivity is much smaller than in the hybrid film. This fits the expectation that the metallic VO_2_ film has a higher impedance mismatch with the surrounding vacuum, which decreases the emission coefficient of the thermal photons.

To have a more direct understanding of the performance, we then calculate the total radiated power (*P*_rad_) of the two samples using the following equation:

(2) $$P_{\mathrm{rad}}(T)=\int d\Omega \cos\theta \int_{2.5\mu m}^{30\mu m}e(\lambda ,\theta )I_{B}(\lambda ,T)d\lambda ,$$

where *e*(*λ*,*θ*) is the emissivity as a function of wavelength *λ* and emission angle *θ*, and *I*_B_(*λ*,*T*) is the spectral blackbody radiance. Because of the thermal hysteresis nature of VO_2_, *e*(*λ*,*θ*) is also a function that is dependent on the temperature history. It can be approximated as follows [29]:

(3) $$\begin{array}{ll} e(\lambda ,\theta ) & =e_{m}(\lambda ,\theta )\cdot \frac{1}{2}\left[1+\mathrm{erf}\left(T-T_{c}\pm \frac{1}{2}\Delta T_{c}\right)\right]\\ & +e_{i}(\lambda ,\theta )\cdot \frac{1}{2}\left[1-\mathrm{erf}\left(T-T_{c}\pm \frac{1}{2}\Delta T_{c}\right)\right], \end{array}$$

where the subscript m and i denote the metallic and insulating VO_2_, respectively, erf is the error function used to simulate the hysteresis, *T*_c_ is the temperature point at which the phase change is complete, Δ*T*_c_ is the hysteretic width, and signs ± correspond to the cooling and heating process of the loop, respectively. Working according to the previous experimental results [30,31], we set *T*_c_ = 340 K and Δ*T*_c_ = 10 K.

**Figure 5:**
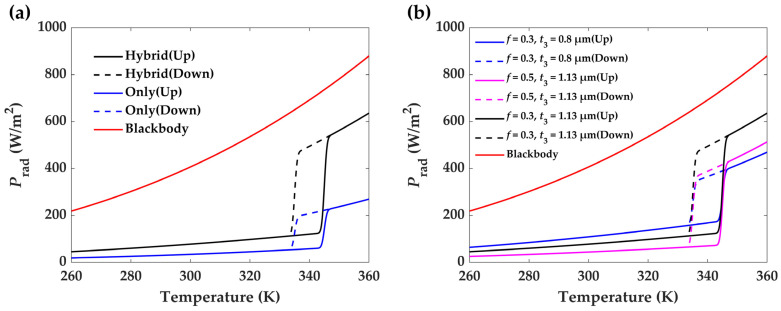
Thermal radiation power of the samples at different temperatures. (**a**) The radiation response of the optimized structures for both hybrid and VO_2_ films. (**b**) The thermal radiation properties of the hybrid film structure at different combinations of the filling factor *f* and the film thickness *t*_3_. Hysteresis behavior is exhibited by solid and dashed lines, respectively, representing the heating and cooling process. The blackbody radiation (red solid) is also given for comparison.

Taking the results in Figure 4 and using all parameters in Equation (2), we obtain the radiated power (*P*_rad_) as a function of the operation temperature (Figure 5a). Both samples show a dramatic rise in the radiation power when VO_2_ experiences an insulating–metallic phase transition. However, the power amplitude for the hybrid film is much larger than that of the pure VO_2_ film case. The hysteresis behavior of the power curve is determined by the VO_2_ phase state, which is amplified by optimizing a proper structural design. Meanwhile, the loop area corresponds to the energy consumed in the phase change process. It is key to note that temperature’s historical dependence on emission power is highly beneficial for enhancing the transient response of the cooling system, which also establishes a narrow temperature window in homeostasis occurs. Reading from the curves, we observe that the hybrid sample has a radiation power that varies from 114 to 542 W/m^2^ in the temperature window of 340 ± 7 K, while it is 51–226 W/m^2^ for the VO_2_ reference. At the thermal equilibrium state, a higher thermal regulation performance is expected for the former.

When designing a homeostasis system, the main requirement is to have a large difference in radiation power between the two VO_2_ states. This is primarily exhibited by the emissivity. Because of the angular dependence of emissivity in a film’s structure, mathematically, it is not proper to use the emissivity as the target function in parametric optimization. Alternatively, we directly set the radiation power difference at the inflection point of the heating and cooling processes (Figure 5a) and use the filling factor (*f*) and the film thickness (*t*_3_) as the variables in the optimization procedure. Considering the realistic situation, we set the lower limit of *t*_3_ to 1 μm. Applying the PSO algorithm, the optimal combination we obtain is *f* = 0.3 and *t*_3_ = 1.13 μm, which is reasonable because the maximum value of the filling factor is 0.74 (cubic closest packing). Figure 5b shows the comparison results when different values are selected for the two variables. The optimal combination can give rise to the largest difference in radiation power.

## 3. Results and Discussion

After optimizing the hysterical radiation power loop, we now go on to inspect its temperature regulation performance under an external heat pump with varying powers. To illustrate the idea, we calculate the transient thermal behavior of the two samples. Using an input of a square heat power source. To mimic real skin, we assume that the bionic coat covers a body object made of 0.5 mm thick silicone. In the calculation, it is assumed that the covered object can be treated as isothermal and isolated from air and water vapor, which implies that the input power (*P*_in_) is entirely directed towards the sample structure. Under this assumption, the equilibrium temperature of the sample is mutually decided by the input power and radiated power using the following equation [29]:

(4) $$\rho CL_{c}\frac{dT(t)}{dt}=P_{\mathrm{in}}(t)-P_{\mathrm{rad}}(T),$$

where ***ρ*** is the mass density, *C* is the heat capacitance, and *L*_c_ is the characteristic length scale (or the penetration depth of the conduction heat flow). To simplify the expression, we rescale the time variable

(5) $$t_{e}=\frac{t}{\rho CL_{c}},$$

after which Equation (4) becomes the following:

(6) $$\frac{dT\left(t_{e}\right)}{dt_{e}}=P_{\mathrm{in}}\left(t_{e}\right)-P_{\mathrm{rad}}(T).$$

**Figure 6:**
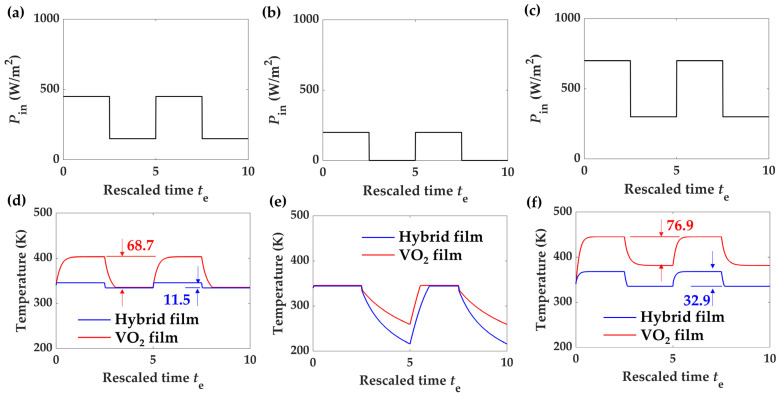
Temperature fluctuation at different input powers for the samples. The input power is 150–450 W/m^2^ for (**a**), 0–200 W/m^2^ for (**b**), and 300–700 W/m^2^ for (**c**), and the corresponding transient temperature responses for the hybrid sample (blue solid) and the VO_2_ reference (red solid) are given in (**d**), (**e**), and (**f**), respectively. In (**d**,**f**), the temperature fluctuation range at the homeostasis is also added.

Using the above equation, we calculate the transient response with dynamic input power. Firstly, we consider the heat load with the power varied between 150 and 450 W/m^2^, as plotted in Figure 6a, which matches the radiated power range of the hysteresis loop discussed before. The results calculated for the two sample structures are shown in Figure 6d. Within this power range of the heat load, the hybrid film sample shows a temperature change of Δ*T* = 11.5 K, and for the VO_2_ film sample, the value is Δ*T* = 68.7 K. Additionally, the hybrid film not only reduces the temperature fluctuation range but also responds more than 16 times faster than the pure VO_2_ film, which ensures its feasibility in protecting devices which need a stable working temperature. However, the device will lose its self-adaptive temperature regulation capability when the heat load is out of the desired range. For example, when the square input power chooses 0–200 W/m^2^ (Figure 6b), the output temperature merely reflects a cooling process. For the sample coated by the hybrid film, this process is much faster due to its larger emissivity (Figure 6e). On the other side, with higher input powers, for example, as plotted in Figure 6c with *P*_in_ = 300–700 W/m^2^, the transient response time is lengthened, and the temperature fluctuation increases to 32.9 K for the hybrid film and 76.9 K for the VO_2_ film.

The permissible heat power load is determined using the height of the hysteresis loop, as shown in Figure 5a. As illustrated before, the desired input (114–542 W/m^2^) is obtained by considering the fabrication complexity of the current hybrid film and whether it can satisfy certain practical conditions, such as the need for the energy scale to be comparable with normal body radiation. If needed, it is fully possible to broaden the power range at the cost of structural complexity. On the other hand, reducing the width of the hysteresis loop of VO_2_ can help to narrow the final homeostasis temperature range, which can be realized via fabrication [31]. It is already known from material sciences that the phase change temperature *T*_c_ or the working temperature of the skin coat can be reduced by doping elements like tungsten [32,33]. In principle, a smart skin, with homeostasis occurring around room temperature, can be expected. This could have more practical applications.

**Figure 7:**
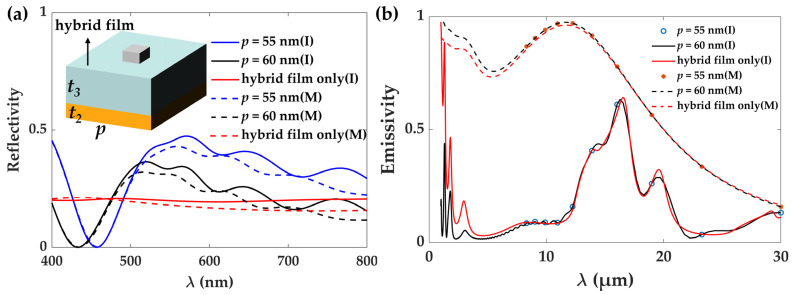
Visible and infrared spectra properties. (**a**) Reflectivity in the visible light spectra at various points of the silver nanocube. The inset gives one unit of the silver nanocube, comprising the top plasmonic surface. (**b**) Emissivity properties of the infrared spectrum. The normal light incidence and emissions are calculated here.

In the above, we design an artificial bionic skin to realize self-adaptive temperature regulation by optimizing the infrared spectrum. For real applications, the visible light’s appearance is also relevant. The designed skin itself looks black due to the large loss in the visible band, no matter whether it has metallic or insulating VO_2_ inclusions. To solve the problem, we introduce a plasmonic surface made of silver nanocubes upon the hybrid film, as shown in the inset of Figure 7a. The side length and thickness of nanocubes are fixed at 40 nm and 50 nm, respectively, which are classic sizes. These are achieved via a chemical sell-assembling process [34]. The period (*p*) of the nanocubes can be utilized to tune the color features of the sample. Because of the plasmonic response of the silver particles, the nanostructures can strongly interact with the incident light and induce selective spectral reflectance. Additionally, the metallic surface with a tiny period is nearly transparent in the far-infrared spectrum and thus has no impact on optimized cooling properties, as shown in Figure 7b. Here, we give just one simple case to prove the possibility of manipulating the color of the artificial skin. Different nanostructures can be adopted to develop more versatile control of the visible light properties. This has been heavily explored before to set structural colors [35,36,37].

## 4. Conclusions

In this work, we propose the development of a smart skin capable of self-adaptive temperature regulation [38,39] through the utilization of phase-changing properties of VO_2_. When our hybrid film is applied to a silicone substrate, it can maintain temperatures around 340 ± 6 K under varying external heat fluxes ranging from 150 to 450 W/m^2^. This temperature fluctuation is significantly less than one-fifth of that observed when the substrate is covered with an optimized pure VO_2_ film. Furthermore, to enhance the spectral similarity to natural skin, we integrate a plasmonic surface composed of self-assembled silver nanocubes, modifying the visible light properties of the bionic skin while allowing it to retain its infrared characteristics. Although it is relatively straightforward to fabricate artificial structures with selective multiband spectral features through physical methods such as multilayer techniques, the challenge intensifies when integrating multiple factors, including varying operational states (in this case, different temperatures), topology, reproducibility, and cost considerations. We contend that our research exemplifies an effective approach to creating a smart temperature-responsive skin that can address these critical challenges in practical applications.

## Data Availability

The original contributions presented in the study are included in the article, further inquiries can be directed to the corresponding author.
